# Dental and Maxillofacial Manifestations of Axenfeld–Rieger Syndrome: Presentation of a Case in a 5-Year-Old Girl

**DOI:** 10.1155/2022/4348264

**Published:** 2022-08-02

**Authors:** Carlos-Martín Ardila, Efraín Álvarez-Martínez

**Affiliations:** Universidad de Antioquia, Medellín, Colombia

## Abstract

In this case study, the dental and maxillofacial manifestations of a 5-year-old girl with Axenfeld–Rieger syndrome (ARS) are described. This syndrome is characterized by craniomaxillofacial, ocular, dental, and umbilical disorders. The patient presented ocular dyscoria and corectopia, iris abnormalities, midface hypoplasia with a thin upper lip, and a protruded lower lip. Hyperplastic maxillary labial frenulum, oligodontia, ghost teeth, bilateral Class III molar and canine relationship, and right posterior crossbite were also found. An everted umbilicus with redundant periumbilical skin was evident. Early diagnosis of ARS is essential to establishing preventive and corrective measures that provide a good quality of life for patients who suffer from this syndrome.

## 1. Introduction

Axenfeld–Rieger syndrome (ARS) is a rare autosomal dominant disorder with a worldwide incidence of 1 : 200,000. It encompasses a spectrum of genetically inherited disorders that are characterized by dental, ocular, craniofacial, and periumbilical abnormalities [1]. It is a hereditary disorder with almost complete penetrance and variable expression. Inheritance is autosomal dominant in 70% of cases [2]. In addition to its pathogenesis, there are no definitive data on its racial or gender prevalence. It is believed to arise as an ectodermal tissue defect caused by disorders of the neural crest. The differentiation and migration of the neural crest cells are responsible for (among other things) the induction of the enamel organ from the oral epithelium, and for the formation of normal ocular and craniofacial structures [3]. ARS has been associated with a wide variety of oral and dental abnormalities, including deciduous and permanent hypodontia (particularly maxillary anteriors), hyperplastic maxillary labial frenulum, microdontia, taurodontia, enamel hypoplasia, and peg-shaped teeth. Craniofacial features are dominated by midface hypoplasia due to maxillary hypoplasia, relative mandibular prognathism, and a broad, flat nasal root. Moreover, protruded lower lip or recessive upper lip and prominent supraorbital ridges are observed [2, 4].

Three types of ARS have been described. Type 1 is mainly characterized by dental abnormalities and midface hypoplasia secondary to underdeveloped maxillary sinuses. Type 2 includes hearing loss and heart defects, while Type 3 features ocular manifestations but lesser facial and dental abnormalities [5]. Recently, important innovations in the molecular genetics of ARS and associated phenotypes have been described. Three genetic loci have been recognized on chromosomes 4q25, 6p25, and 13q14. The genes at 4q25 and 6p25 have also been acknowledged and are termed “PITX2” and “FKHL7”, respectively. The detection of these loci and genes has shed novel information on the interrelation of the Axenfeld–Rieger phenotypes. Therefore, all of these phenotypes may be considered together under the single heading of ARS, with the benefit of simplifying interaction between clinicians and scientists and remove subjective and unclear subclassification [6].

This article describes the maxillofacial and dental characteristics of one of the few cases worldwide of ARS that have been reported.

## 2. Case Report

A 5-year-old girl, accompanied by her father, consulted for her daughter's dental alterations. The patient did not report any pain. The medical history revealed a consult for pupillary dyscoria, with no family history of craniofacial or dental anomalies. The patient's diagnosis of ARS was made during the first years of her life.

In the general examination, the patient had a good disposition, she was oriented in time, place, and person, and communicated easily.

**Figure 1:**
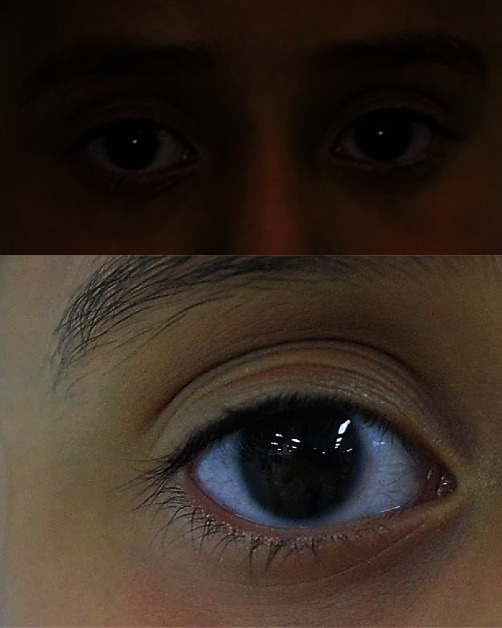
Evidence of pupillary dyscoria.

**Figure 2:**
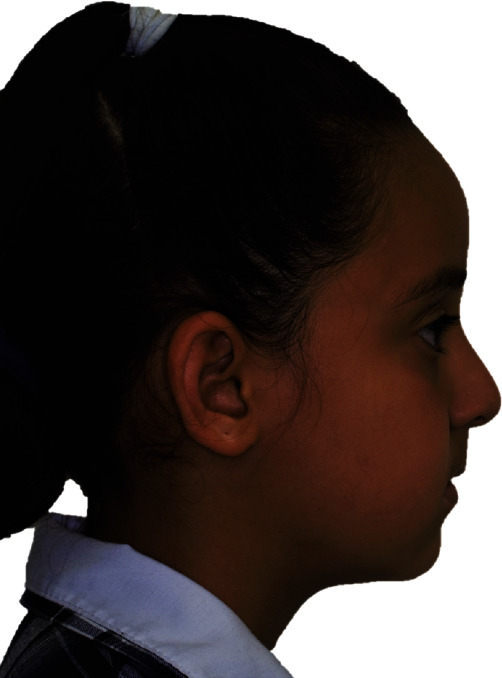
There is a greater predominance of the upper third of the face with respect to the middle and lower thirds. A slightly flattened mentolabial angle is observed.

Her extraoral characteristics included pupillary dyscoria (Figure 1), corectopia, blepharitis, abnormalities in the iris, and posterior embryotoxon. In profile analysis, there is a greater predominance of the upper third of the face with respect to the middle and lower thirds (Figure 2). Moreover, a slightly flattened infraorbital area, prominent cheekbones, straight nasolabial angle, superior retrochelia, resting labial seal, inferior normochelia, and slightly flattened mentolabial angle are observed (Figure 2).

**Figure 3:**
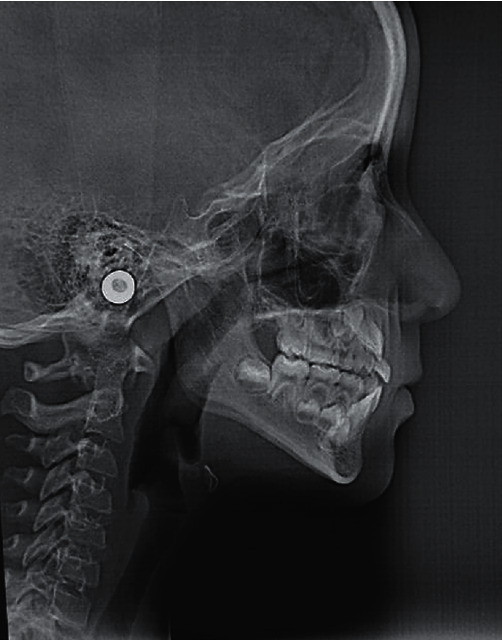
The lateral cephalic radiography shows skeletal Class III due to mild maxillary macrognathism.

In the lateral cephalic radiography, skeletal Class III due to mild maxillary macrognathism is shown. In addition, an increased lower facial third is observed (Figure 3).

**Figure 4:**
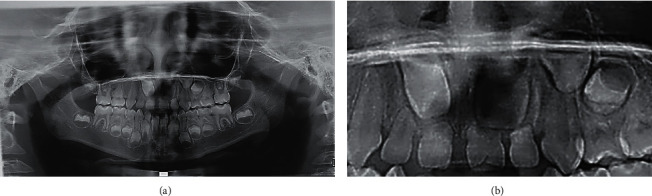
Panoramic X-ray shows teeth 17-11-21-23-24 and 27 in intraosseous formation, in Nolla stage between 3 and 5. The formation of the other maxillary permanent teeth is not appreciated, indicating their agenesis (a and b). Panel (b) shows a close-up view of the anterosuperior area. The formation of teeth 12, 22, and 13 is not appreciated.

**Figure 5:**
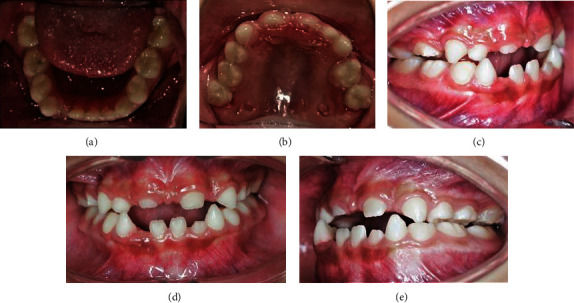
Asymmetrical oval arches and dental malocclusion Class III are shown (a and b). Traction upper labial frenulum associated with lack of vertical development of the superior alveolar process is observed (c, d, and e).

The panoramic X-ray (Figure 4) shows a slightly narrower right nostril than the left. Early mixed maxillary dentition is observed where 16 and 26 are erupted. Teeth 17-11-21-23-24 and 27 are in intraosseous formation, in Nolla stage between 3 and 5; therefore, the formation of the other maxillary permanent teeth is not appreciated, indicating their agenesis (Figure 4). The patient presents mixed mandibular dentition (Figures 4 and 5); it is also observed that 36-31-41-46 erupted without apical closure (physiological phase), complete deciduous dentition, except 71 and 81 already exfoliated (Figure 4).

Intraorally, traction of the upper labial frenulum, associated with lack of vertical development of the superior alveolar process, is observed. Asymmetric oval arches and dental malocclusion Class III are shown (Figure 5).

**Figure 6:**
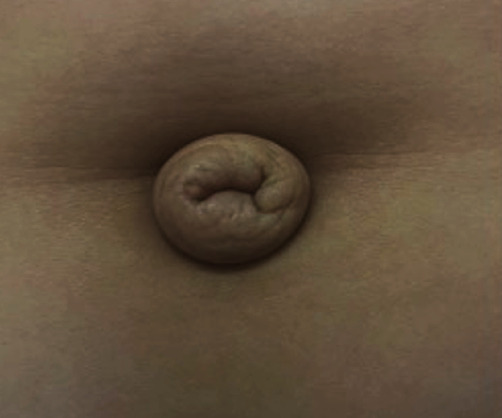
The existence of an everted umbilicus.

Another prominent manifestation was the everted umbilicus with redundant periumbilical skin (Figure 6).

To ensure the preservation of the existing dentition, preventive dental measures were recommended, including oral hygiene instruction to the girl and her parents, as well as the sealing of pits and fissures of all molars. Trimestral check-ups with the dentist were also suggested. The orthodontist recommended a facial mask protocol with a Hyrax and a posteroinferior bite plane. The Hyrax was recommended to prevent cross-sectional discrepancy between the arches considering the Class III relationship and dental agenesis, in addition to disarticulating the circumpubertal sutures to perform the protraction of the maxilla. The posteroinferior bite plane was recommended to control verticality. The facial mask protracts the maxilla and contains mandibular growth.

## 3. Discussion

In this case, the dental and maxillofacial manifestations of a 5-year-old girl with ARS were described. To the authors' knowledge, this case represents the youngest patient with this syndrome, incorporating reportage of its dental and maxillofacial features. Early dental care of a patient with ARS allows establishing multidisciplinary prevention and treatment strategies that will improve the long-term prognosis of this complex condition. Early detection of ARS can also prevent serious ophthalmological problems.

The literature has extensively described the genetic etiology, ophthalmological and cardiac characteristics, and the treatment of ARS; however, the dental and facial characteristics and their management have been detailed to a lesser extent [7].

The craniomaxillofacial singularities found in ARS commonly include a broad forehead, sella turcica disorders, telecanthus, hypertelorism, dyscoria, disorders of the characteristics of the nose, hypoplasia of the midface, mandibular prognathism, an open-mouth appearance with a thin upper lip and retracted, and with a prominent lower lip [7]. However, some patients do not present any malformation of the craniofacial structures or skeletal bones, and are considered to have a variant of Rieger syndrome called ARS [8]. As in the case reported by Megighian et al. [8], in addition to the presence of dyscoria, the present case exhibits no major craniofacial bone changes, except for hypoplasia of the midface and a pseudoprognathism [5]. It has also been described that maxillary hypoplasia is found in around 90.5% of cases, with a skeletal Class III concave facial profile associated with a flat midface [7].

Oral manifestations include hyperplastic upper labial frenulum, enamel hypoplasia, hypodontia, oligodontia, microdontia, taurodontism, conical teeth, short roots, and delayed eruption [2, 4, 7, 9]. Some of these characteristics were evident in the present case, including that the upper anterior teeth are more frequently absent [8]. In this regard, it has been considered that the number of absent teeth has a specific role in the extent of maxillary hypoplasia due to the resulting alveolar hypoplasia [7, 9].

In this case, like that reported by Agarwal et al. [7], an everted umbilicus with redundant periumbilical skin was evidenced, mimicking an umbilical hernia [2].

Considering the characteristics of the present case, the multidisciplinary management includes the correction of maxillary hypoplasia using myofunctional orthopedic appliances, in addition to preserving the deciduous teeth as far as possible to maintain function and aesthetic appearance. Depending on the evolution of the dental eruption, the assisted eruption of the impacted teeth and the orthodontic correction of the preexisting dentition could be planned [10]. Oligodontia could be treated with prosthetic rehabilitation to restore form and function; dental implant restoration would be a feasible long-term treatment option. Consultation with ophthalmology is essential in order to control the evolution of vision.

Early diagnosis of ARS is essential to establish preventive and corrective measures that provide a good quality of life for patients who suffer from it.

## Data Availability

The clinical data utilized in this report are described in this article.
